# Supplementary material for: Comprehensive Annotation and Functional Exploration of MicroRNAs in Lettuce
Source: Front Plant Sci. 2021 Dec 24;12:781836. doi: 10.3389/fpls.2021.781836 (PMC8739914; doi:10.3389/fpls.2021.781836)
Supplement: Supplementary file 3 [file Data_Sheet_9.ZIP › GSM2230753.plot/Lsa-miRN1650_Lsat_1_v5_gn_9_73101.1_1011_TPlot.pdf]

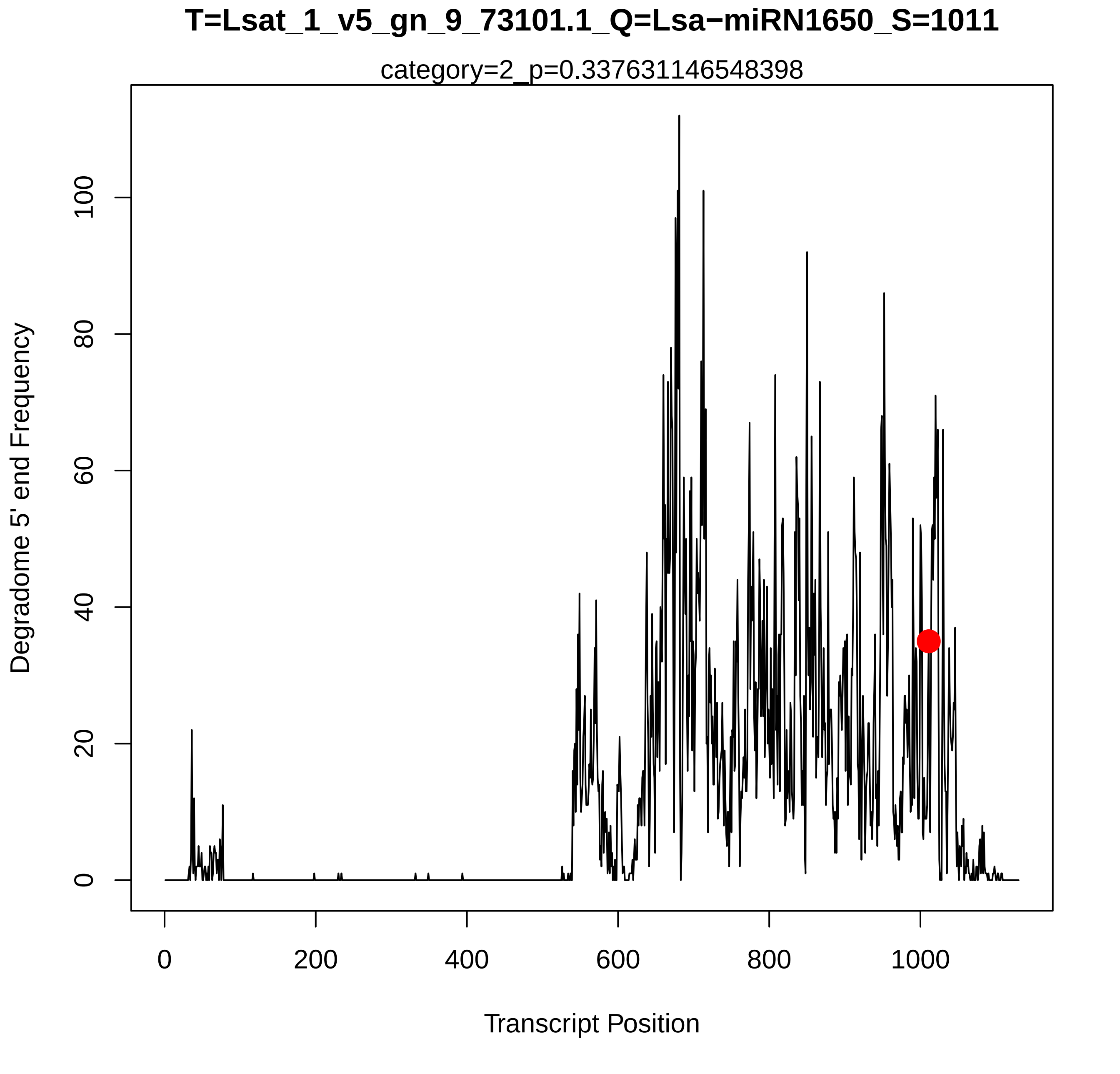
T=Lsat_1_v5_gn_9_73101.1_Q=Lsa−miRN1650_S=1011
category=2_p=0.337631146548398
Degradome 5' end Frequency
0
20
40
60
80
100
0
200
400
600
800
1000
Transcript Position
